# Establishing a quantitative fluorescence assay for the rapid detection of kynurenine in urine†

**DOI:** 10.1039/d2an00107a

**Published:** 2022-03-30

**Authors:** Kamlesh Patel, Marcos Fernandez-Villamarin, Craig Ward, Janet M. Lord, Peter Tino, Paula M. Mendes

**Affiliations:** School of Chemical Engineering, University of Birmingham Edgbaston Birmingham B15 2TT UK p.m.mendes@bham.ac.uk; Institute of Inflammation and Ageing, University of Birmingham Mindelsohn Way Birmingham B15 2TH UK; NIHR Surgical Reconstruction and Microbiology Research Centre, University Hospital Birmingham and University of Birmingham Birmingham B15 2TH UK; School of Computer Science, University of Birmingham Edgbaston Birmingham B15 2TT UK

## Abstract

The kynurenine metabolite is associated with many diseases and disorders, ranging from diabetes and sepsis to more recently COVID-19. Here we report a fluorescence-based assay for the detection of kynurenine in urine using a specific chemosensor, 3-formyl-4-(ethylthio)-7-(diethylamino)-coumarin. The assay produces a linear response at clinically relevant ranges (1–20 μM), with a limit of detection of 0.7 μM. The average standard addition recoveries of kynurenine in synthetic urine samples are near to 100%, and the relative standard deviation values are less than 8%. The established fluorescence assay for quantitative analysis of kynurenine in urine is facile, sensitive and accurate and holds great potential for low-cost and high-throughput analysis of kynurenine in clinical laboratory settings.

## Abstract 2

A fluorescence-based assay for the detection of kynurenine in urine for low-cost and high-throughput analysis in clinical laboratory settings.

## Graphical abstract

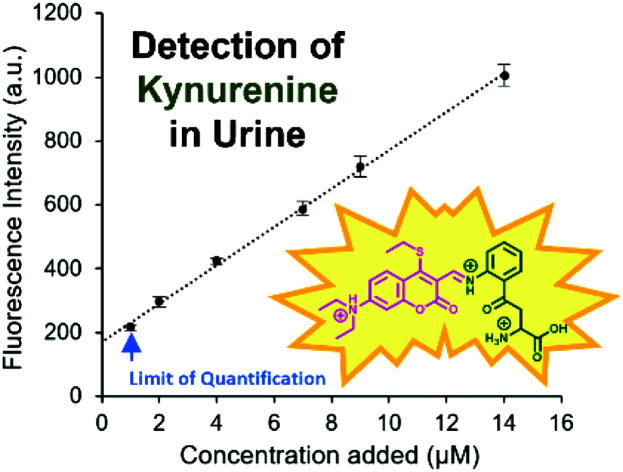

## Introduction

Proteins, glycans and metabolites are important biomarkers for clinical diagnostics.^1–6^ One such biomarker is the metabolite kynurenine, which has been demonstrated to be involved in many diseases and disorders, including diabetes,^7^ coronary artery disease,^8^ invasive tumour growth,^9^ neurological diseases,^10,11^ sepsis,^12,13^ and more recently COVID-19.^14^ The breakdown of the essential amino acid tryptophan towards the production of nicotinamide adenine dinucleotide is known as the kynurenine pathway, and it is closely related to immune activation.^15–17^ Kynurenine is the first metabolite in this pathway and its concentration can therefore be affected by immune responses to disease. Since higher levels of kynurenine are related to a range of medical conditions and have been associated with unfavourable outcomes, it would be advantageous to have a clinically usable quantitative assay to evaluate kynurenine concentrations. In clinical assays, urine or serum are commonly used biological media. In comparison with serum, urine has a lower protein content and more metabolites, so is therefore a less complex matrix to facilitate analytical detection.^18^ In addition, urine is easily obtainable and can be collected frequently and non-invasively in a clinical setting. Here, we have developed a fluorescence-based assay for kynurenine detection in urine that utilises a kynurenine-specific small molecule sensor, creating an effective testing platform to aid clinicians in patient evaluation and prognosis.

Kynurenine detection is often performed using chromatographic methods. High performance liquid chromatography (HPLC) has been used on blood samples,^15,16,19^ as has liquid chromatography coupled with tandem mass spectrometry (LC-MS).^7,8,17^ Gas chromatography has also been employed with tandem mass spectrometry (GC-MS) when analysing urine samples.^7,8^ Whilst chromatographic measurements are sensitive and require low sample volumes, they require lengthy sample preparation alongside large volumes of solvents for column preparation, analyte separation, and rinsing.^19^ On the other hand, mass spectrometric measurements can increase sensitivity and reduce analysis time but requires the use of expensive equipment and highly skilled analysts. Therefore, kynurenine chemosensors would be useful to overcome issues of expense, time and preparation. However, there is limited literature in kynurenine biosensing without the use of chromatographic processes. There has been some research in electroanalytical quantification of kynurenine which has produced sensitive chemosensors and simplified the sample preparation and processing.^20–22^ However, whilst electroanalytical methods can produce sensitive measurements in point of care formats such as glucose sensing, there is still some challenges to address before mass clinical testing.^23^

Ideally, a useful kynurenine sensor would have high-throughput capabilities allowing for rapid results from multiple patient samples. In clinical settings, fluorescence is routinely utilised in formats such as immunoassays, where existing high-throughput technology is available in routine clinical biochemistry laboratories. However, fluorescence-based kynurenine sensors have been barely explored in comparison with other techniques.^24^ A coumarin-based chemosensor was previously shown^25^ to bind kynurenine with high specificity, capable of discriminating kynurenine from other aliphatic and aromatic primary amines. Herein, we implement the use of this chemosensor to establish a fit-for-purpose fluorescence assay for the accurate quantification of kynurenine in urine. The objective is to set a straightforward procedure that can avoid the interference of other biological entities, eliminate intricate purification steps and potentially reduce costs, while opening up the possibility for automatization. For this pursuit, detailed spectroscopic investigations were first performed to achieve high sensitivity and wide linear range, which were subsequently combined with a standard addition method to eliminate the matrix effects of urine samples.^26,27^ Our assay requires non-invasive sampling, small sample volumes, and utilises rapid fluorescence measurements. Furthermore, this technique is easily translatable into clinical laboratories, and could be applied to a wide range of diagnostic settings.

## Materials and methods

### Buffer

A pH 1 buffer was made using hydrochloric acid (HCl) and potassium chloride (KCl). 0.2 M solutions of HCl and KCl were prepared individually. 50 ml 0.2 M KCl was mixed with 134 ml 0.2 M HCl, pH of this mixture was measured using a calibrated pH meter and adjusted by adding concentrated HCl dropwise, then was made up to 200 ml using ultrapure water.

### Solution preparation

Kynurenine was dissolved in the pH 1 buffer or in synthetic urine (Surine™ Negative Urine Control) to the desired concentration. Chemosensor was dissolved in a minimal amount of dimethyl sulfoxide (DMSO), then diluted to the desired concentration by adding pH 1 buffer. In any case, amount of DMSO in final solution never exceeded 1%. Kynurenine and chemosensor solutions were mixed in a 1 : 1 ratio, then the mixture was allowed to rest for one minute for the reaction to occur before measurement.

### Fluorescence and UV-Vis spectroscopy

Fluorescence work was carried out on a combination of a Jasco FP-8500 spectrofluorometer and a BMG Labtech CLARIOstar Plus microplate reader. Both machines use monochromators for selection of the excitation and emission wavelengths. UV-Vis experiments were carried out on either a UV-1800 (Shimadzu) or a Cary 5000 (Varian) spectrophotometer. A combination of quartz and disposable cuvettes were used.

## Results and discussion

**Scheme 1:**
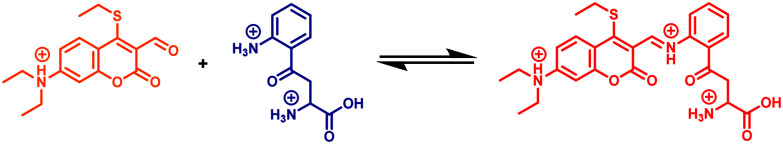
Equilibrium reaction of chemosensor and kynurenine at pH 1.

To develop the spectroscopic kynurenine detection assay 3-formyl-4-(ethylthio)-7-(diethylamino)-coumarin,^25^ a chemosensor that reversibly binds with kynurenine, (Scheme 1) was used. This molecule has an aldehyde moiety that reacts spontaneously with the amine moiety of kynurenine in acid media, forming an imine bond. The resulting compound has an extended conjugation, yielding a redshifted fluorescence that it is easily differentiated from other components.

**Fig. 1:**
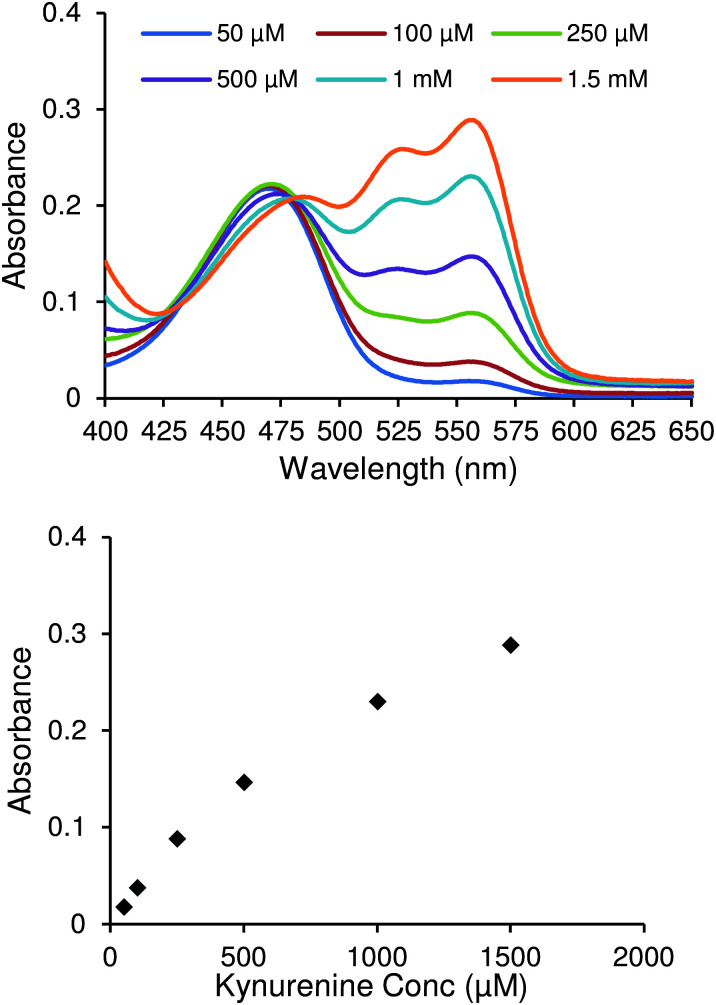
UV-Vis spectra of 10 μM chemosensor solution with varying kynurenine concentrations (top). Absorbance values of same spectra at 555 nm (bottom).

The chemosensor was produced *via* a four-step synthesis following reported procedures (see ESI†), with the final step involving the nucleophilic substitution of 3-formyl-4-(chloro)-7-(diethylamino)-coumarin with ethanethiol. After purification, binding studies with kynurenine were performed and followed by UV-vis spectrophotometry. Fig. 1 shows UV-Vis spectra of the chemosensor after adding increasing concentrations of kynurenine. Two changes ascertained the formation of the chemosensor/kynurenine complex. Firstly, there was a small decrease in the absorbance at 469 nm, which indicated the reduction of free chemosensor in solution. Secondly, the appearance of two new absorbance peaks at 526 nm and 555 nm attributed to complexation with kynurenine. Both of these factors confirmed that this chemosensor could be used in the design of a quantitative kynurenine assay.

From these results, it was clear that direct application of UV-Vis methodology alone for the quantification of kynurenine was not sensitive enough for the required range of detection. The lowest kynurenine concentration studied (50 μM) corresponds to an absorbance value around 0.04. Expected levels of kynurenine would be between 2–6 μM (ref. 12) and 1.4–3.8 μM (ref. 8) in blood and urine, respectively. Thus, it is difficult to precisely detect concentrations in clinically relevant ranges with high sensitivity using an UV-Vis methodology due to its low response. Fluorescence techniques, however, provide superior absolute responses, and thus were used in further studies.

**Fig. 2:**
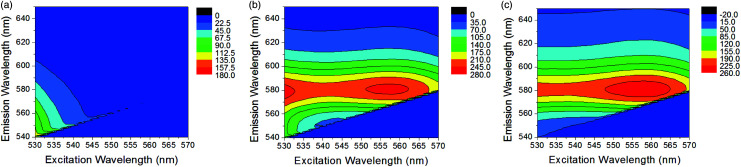
3D fluorescence spectra of 10 μM chemosensor (a), 10 μM chemosensor with 6 μM kynurenine (b) and their difference (c). The colour map shows fluorescent intensity values with greater intensity values being a darker red colour.

Following confirmation of successful chemosensor-kynurenine binding, it was important to optimise the excitation and emission wavelength parameters to build a sensitive fluorescence assay. Measurements performed with chemosensor alone (10 μM) and mixed with kynurenine (6 μM) are shown in Fig. 2. 3D fluorescence spectra were measured using excitation wavelengths between 530–570 nm and recording emission intensity between 540–650 nm. In this range, the chemosensor (Fig. 2a) presented its own fluorescence that overlapped with complex fluorescence (Fig. 2b). A subtraction between both graphs was performed to obtain uncontaminated complex fluorescence (Fig. 2c). This operation allowed the elimination of secondary maximum peaks unrelated to the compound of interest. It was determined that using an excitation wavelength of 560 nm and an emission wavelength of 580 nm were optimal conditions for creating a sensitive kynurenine binding assay. The choice of these wavelengths also assures the selective measurement of the chemosensor-kynurenine product. This chemosensor works on the principle that an unprecedented very large bathochromic shift occurs when its functional aldehyde reacts with the aromatic amine in kynurenine. Real urine contains a series of metabolites such as amino acids and adenosine derivatives, which are present at concentration ranges of those found for kynurenine.^28,29^ Based on their chemical structures, similar behaviour as previously observed for other non-target related amines, namely glycine, adenosine and cytidine, is expected.^25^ While they will bind to the sensor, the bathochromic shifts associated with their binding are very small compared to that observed for the kynurenine sensor product. Thus, the unique spectral characteristics of the kynurenine sensor allows for the precise discrimination of kynurenine in complex mixtures containing other amine derivatives, as those encountered in urine.

**Fig. 3:**
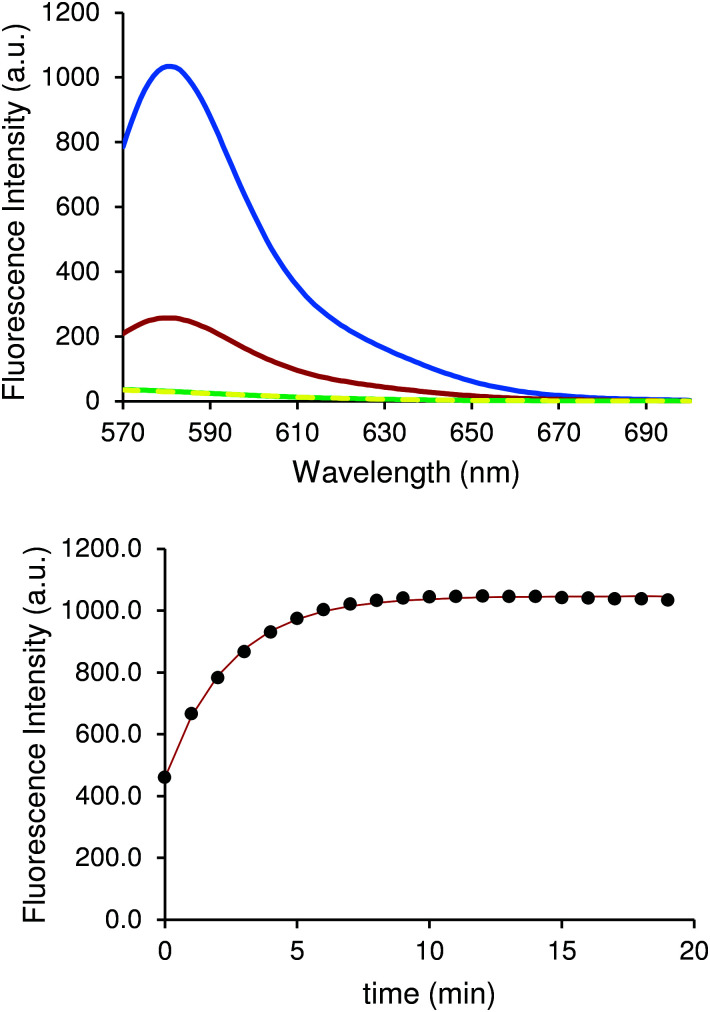
Fluorescence spectra of 50 μM chemosensor solution at 10 °C (yellow) and 50 μM chemosensor with 6 μM kynurenine at 10 °C (blue), 30 °C (red) and 90 °C (green) (top). Variation of fluorescence intensity at 580 nm of a 50 μM chemosensor solution with 6 μM kynurenine from room temperature (approx. 22 °C) after being subjected to 10 °C (bottom). The solution was given 20 minutes to equilibrate to each temperature before each spectra were recorded.

Once optimal excitation and emission wavelength parameters were selected, it was important to investigate the effect of temperature on the sensitivity of our assay, since this can have an effect on fluorescence due to equilibrium reactions. Three different experiments were set at 10 °C, 30 °C and 90 °C and allowed to reach equilibrium for 20 min (Fig. 3), after which kynurenine was added. The fluorescence output showed a large variation depending on the temperature at which the experiment was performed. The intensity measured at the emission maximum (580 nm) increased four-fold when the temperature was lowered from 30 °C to 10 °C.

This behaviour is due to the displacement of reaction equilibrium towards products, which is the analyte of the measurement. The same solution heated to 90 °C translated into an eightfold decrease in fluorescence intensity measured at the emission maximum (580 nm). In fact, this experiment showed no difference when compared with spectra of chemosensor solution. Almost identical signals for both cases can be seen in Fig. 3, demonstrating that as the temperature increases, the equilibrium shifts towards the reactants. Experiments conducted at 10 °C showed optimal results and subsequent experiments were performed at that temperature. Many instruments used to measure fluorescence often incorporate active heating/cooling systems allowing for specific control to maximise signal output.

Once we had tailored the reaction conditions for optimal fluorescence output, we investigated the capability of our assay for detecting clinically relevant concentrations of kynurenine. The range of chemosensor concentrations was determined by measuring the fluorescence response with increasing kynurenine concentrations. Urine levels of kynurenine are determined to be in 1.4–3.8 μM,^8^ so the range of kynurenine concentration was set between 1–20 μM.

Quantification of kynurenine was achieved by measuring the product of a reversible reaction with the chemosensor. Three concentrations of chemosensor in a similar range as kynurenine were selected, specifically 1, 10, and 20 μM, to evaluate the linearity of the response.

**Fig. 4:**
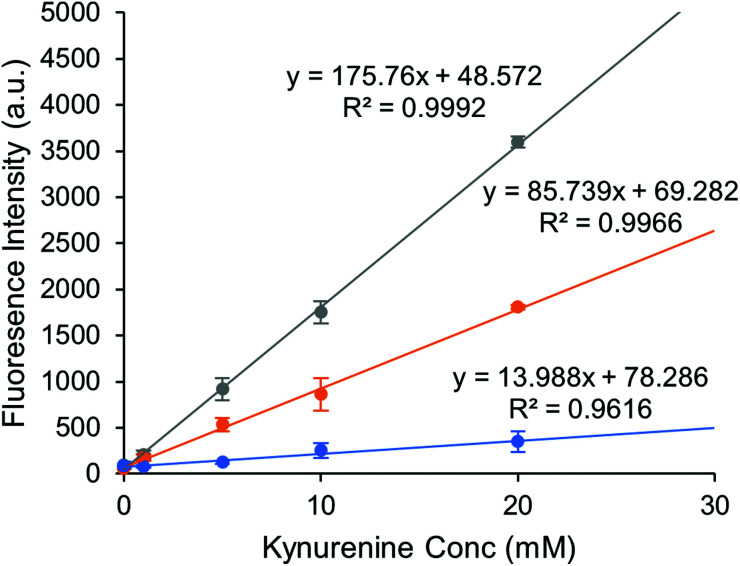
Fluorescence intensity of 1 μM (blue), 10 μM (orange) and 20 μM (grey) chemosensor solutions with different kynurenine concentrations, measured at optimal conditions. The graph plots the average of 3 measurements with error bars of 1 standard deviation. Linear regression was performed considering each replicate as a separate data point.

Fig. 4 shows the results when using chemosensor concentrations of 1, 10, and 20 μM with 1–20 μM concentrations of kynurenine. The graph shows that the fluorescence output was directly proportional to kynurenine concentration, creating a simple and effective assay. All chemosensor concentrations enabled an effective measurement of kynurenine at the physiological levels expected to be obtained in real biological samples.

Selection of the best chemosensor concentration to perform further sample analysis was decided by limit of detection (LOD, 3.3*σ*/*S*) and limit of quantification (LOQ, 10*σ*/*S*). By fitting the data in Fig. 4, the LOD and LOQ for each chemosensor concentration was determined and displayed in Table 1. The lowest LOD is displayed by 20 μM chemosensor concentration, which allowed detection of less than 1 μM kynurenine. However, since kynurenine levels in clinical urine samples can be still below the LOQ, we took a different approach. The standard addition method was next investigated to decrease inaccuracies in the measurements at lower levels, especially with a view to using a more complex medium.

**Table 1:** Limit of detection and limit of quantification

Chemosensor (μM)	LOD (μM)	LOQ (μM)
1	6.1	18.6
10	1.4	4.3
20	0.7	2.0

**Fig. 5:**
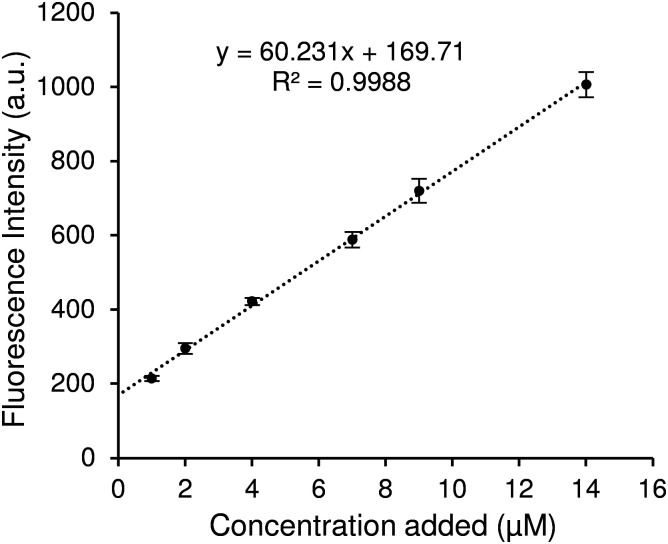
Fluorescence intensity of successive standard additions to a 1 μM synthetic urine solution. The data plotted are the average fluorescence values across three repeats with the error bars set to the standard deviation. Linear regression was performed considering each replicate as a separate data point.

In an attempt to improve the sensitivity of our assay at lower kynurenine concentrations, we used the standard addition method. With the intention of using this assay in complex biological media, the feasibility of this quantification method for kynurenine was assessed with solutions in synthetic urine. Surine™ was selected because it mimics the characteristics of normal human urine, representing a reliable biological matrix for simulating clinical samples.^30–32^ The standard addition method was performed by adding 1, 2, 4, 7, 9 and 14 μM standard kynurenine to a test solution containing 1 μM kynurenine. After performing measurements in optimal conditions, results are shown in Fig. 5. The standard curve slope is lower than observed in Fig. 4, suggesting some effect of the synthetic urine, but all data follow a linear behaviour except the test solution, as its concentration is lower than LOQ. In order to obtain more information from this data, the calibration curve in Fig. 5 was employed to calculate the found concentrations in Table 2. The recovery values were calculated as percentages with respect to the corresponding total known concentrations.

**Table 2:** Determination of kynurenine in synthetic urine samples

Original (μM)	Added (μM)	Total (μM)	Found (μM)	Recovery (%)	RSD (%) (*n* = 3)
1	1	2	1.74	87.3	7.1
1	2	3	3.09	103.0	7.8
1	4	5	5.19	103.8	3.1
1	7	8	7.95	99.4	4.5
1	9	10	10.14	101.4	5.3
1	14	15	14.89	99.3	3.8

As shown in Table 2, recovery for the first addition is rather low (87.3%), however successive standard additions improved recovery, ranging between 99.3 and 103.8%. In all cases, relative standard deviation (RSD) is lower than 8.0%, displaying a good accuracy.

Currently, quantitative analysis for kynurenine in biological samples has been reported using HPLC,^15,16,19^ GC-MS^7,8^ and LC-MS.^7,8,17^ An ultrahigh performance liquid chromatography–electrospray ionization tandem mass spectrometry (UHPLC-ESI-MS/MS) method has been established that can detect kynurenine in urine with a LOD < 11.5 nM.^23^ Urinary concentrations of kynurenine are found at low micromolar levels (>1.4 μM).^8^ Thus, LC-MS-based approaches offer specificity and sensitivity, which is well below the levels required, but it is not without limitations. The processes are medium- to low-throughput, requires highly skilled technicians and time-consuming and costly sample preparation. Electroanalytical methods have been also combined with LC to reach LOD < 35 nM.^33^ Electroanalytical methods enhance the high-throughput capabilities but currently still require both chromatographic and pre-treatment steps for reducing interference from other species in complex media.^23^ Comparing our fluorescence-based approach with currently available alternatives shows that our approach delivers selectivity and enough sensitivity (LOD 0.7 μM) to quantify kynurenine at physiological concentrations in urine samples, without the need for the time-consuming and costly sample preparation. The developed fluorescence-based approach is distinguished by its simplicity, low cost and speed and suitability for automation, enabling high-throughput measurements of large number of samples.

## Conclusions

In this study, different spectroscopic techniques (UV-vis Absorbance and Fluorescence) were evaluated to allow detection of kynurenine in biological samples (urine). Absorbance was limited in its capability to detect low biological concentrations. However, fluorescence was determined to be a suitable alternative, wherein kynurenine-chemosensor binding and associated fluorescence intensity is shown to increase markedly as the temperature decreases. By setting the assay at 10 °C, a linear response at clinically relevant ranges (1–20 μM) is obtained, with a limit of detection of 0.7 μM. After verifying the proper linearity and detection limits of the method, a standard addition strategy was implemented to assure good precision and accuracy. Following that success, synthetic urine was utilised as a mimic of patient samples. The results exhibit similar recovery values as previous proving the accuracy of this method and is even able to correct the low precision shown at low concentrations. The fluorescence assay for kynurenine detection is simple, rapid, and with high throughput capabilities, making it easily translatable into clinical laboratories to assist with diagnostic procedures.

## Author contributions

K. P., M. F.-V. and C. W. conducted the experiments, analysed the data and wrote the manuscript. P. M. M., J. M. L. and P. T. conceived and designed the study and wrote the manuscript.

## Conflicts of interest

There are no conflicts to declare.

## Supplementary Material

AN-147-D2AN00107A-s001

## References

[cit1] Wu L., Qu X. (2015). Chem. Soc. Rev..

[cit2] Scott E., Munkley J. (2019). Int. J. Mol. Sci..

[cit3] Tommasone S., Tagger Y. K., Mendes P. M. (2020). Adv. Funct. Mater..

[cit4] Wang X., Chen G., Zhang P., Jia Q. (2021). Anal. Methods.

[cit5] Garg M., Sharma A. L., Singh S. (2021). Biosens. Bioelectron..

[cit6] Thomas D., Rathinavel A. K., Radhakrishnan P. (2021). Biochim. Biophys. Acta, Rev. Cancer.

[cit7] Rebnord E. W., Strand E., Midttun Ø., Svingen G. F. T., Christensen M. H. E., Ueland P. M., Mellgren G., Njølstad P. R., Tell G. S., Nygård O. K., Pedersen E. R. (2017). Diabetologia.

[cit8] Pedersen E. R., Svingen G. F. T., Schartum-Hansen H., Ueland P. M., Ebbing M., Nordrehaug J. E., Igland J., Seifert R., Nilsen R. M., Nygård O. (2013). Eur. Heart J..

[cit9] Opitz C. A., Litzenburger U. M., Sahm F., Ott M., Tritschler I., Trump S., Schumacher T., Jestaedt L., Schrenk D., Weller M., Jugold M., Guillemin G. J., Miller C. L., Lutz C., Radlwimmer B., Lehmann I., Von Deimling A., Wick W., Platten M. (2011). Nature.

[cit10] Stone T. W., Forrest C. M., Darlington L. G. (2012). FEBS J..

[cit11] Stone T. W., Forrest C. M., Stoy N., Darlington L. G. (2012). J. Neural Transm..

[cit12] Lögters T. T., Laryea M. D., Altrichter J., Sokolowski J., Cinatl J., Reipen J., Linhart W., Windolf J., Scholz M., Wild M. (2009). Shock.

[cit13] van Engelen T. S. R., Wiersinga W. J., Scicluna B. P., van der Poll T. (2018). Crit. Care Clin..

[cit14] Lionetto L., Ulivieri M., Capi M., De Bernardini D., Fazio F., Petrucca A., Pomes L. M., De Luca O., Gentile G., Casolla B., Curto M., Salerno G., Schillizzi S., Torre M. S., Santino I., Rocco M., Marchetti P., Aceti A., Ricci A., Bonfini R., Nicoletti F., Simmaco M., Borro M. (2021). Biochim. Biophys. Acta, Mol. Basis Dis..

[cit15] Tan V. X., Guillemin G. J. (2019). Front. Neurosci..

[cit16] Darcy C. J., Davis J. S., Woodberry T., McNeil Y. R., Stephens D. P., Yeo T. W., Anstey N. M. (2011). PLoS One.

[cit17] de Vries L. V., Minović I., Franssen C. F. M., van Faassen M., Sanders J. S. F., Berger S. P., Navis G., Kema I. P., Bakker S. J. L. (2017). Am. J. Physiol. Renal Physiol..

[cit18] Lima A. R., Bastos M. D. L., Carvalho M., Guedes de Pinho P. (2016). Transl. Oncol..

[cit19] Sadok I., Gamian A., Staniszewska M. M. (2017). J. Sep. Sci..

[cit20] Karami P., Majidi M. R., Johari-Ahar M., Barar J., Omidi Y. (2017). Biosens. Bioelectron..

[cit21] Kato D., Kamata T., Sumimoto M. (2021). Electroanalysis.

[cit22] Sadok I., Tyszczuk-Rotko K., Mroczka R., Staniszewska M. (2020). Talanta.

[cit23] Sadok I., Staniszewska M. (2021). Sensors.

[cit24] Ungor D., Horváth K., Dékány I., Csapó E. (2019). Sens. Actuators, B.

[cit25] Klockow J. L., Glass T. E. (2013). Org. Lett..

[cit26] Mamián-López M. B., Poppi R. J. (2013). Anal. Chim. Acta.

[cit27] Mathaweesansurn A., Thongrod S., Khongkaew P., Phechkrajang C. M., Wilairat P., Choengchan N. (2020). Talanta.

[cit28] Bouatra S., Aziat F., Mandal R., Guo A. C., Wilson M. R., Knox C., Bjorndahl T. C., Krishnamurthy R., Saleem F., Liu P., Dame Z. T., Poelzer J., Huynh J., Yallou F. S., Psychogios N., Dong E., Bogumil R., Roehring C., Wishart D. S. (2013). PLoS One.

[cit29] Dunstan R. H., Sparkes D. L., Macdonald M. M., De Jonge X. J., Dascombe B. J., Gottfries J., Gottfries C. G., Roberts T. K. (2017). Nutr. J..

[cit30] McCulloch R. D., Robb D. B. (2017). Anal. Chem..

[cit31] Huttanus H. M., Vu T., Guruli G., Tracey A., Carswell W., Said N., Du P., Parkinson B. G., Orlando G., Robertson J. L., Senger R. S. (2020). PLoS One.

[cit32] Janyasupab M., Liu C.-W., Chanlek N., Chio-Srichan S., Promptmas C., Surareungchai W. (2019). Sens. Actuators, B.

[cit33] Brooks E. L., Mutengwa V. S., Abdalla A., Yeoman M. S., Patel B. A. (2019). Analyst.

