# Supplementary material for: Establishing a quantitative fluorescence assay for the rapid detection of kynurenine in urine
Source: Analyst. 2022 Mar 30;147(9):1931–6. doi: 10.1039/d2an00107a (PMC9063859; doi:10.1039/d2an00107a)
Supplement: AN-147-D2AN00107A-s001 [file AN-147-D2AN00107A-s001.pdf]

Electronic Supplementary Material (ESI) for Analyst.
This journal is © The Royal Society of Chemistry 2022

Supporting Information for

Establishing a quantitative fluorescence assay for the rapid detection of kynurenine in urine.

Kamlesh Patel[a], Marcos Fernandez-Villamarin[a], Craig Ward[a], Janet M. Lord[b,c], Peter Tino[d] Paula M. Mendes[a,]*

[a] *School of Chemical Engineering, University of Birmingham, Edgbaston, Birmingham B15 2TT, UK.*
[b] *Institute of Inflammation and Ageing, University of Birmingham, Mindelsohn Way, Birmingham B15 2TH, UK.*
[c] *NIHR Surgical Reconstruction and Microbiology Research Centre, University Hospital Birmingham and University of Birmingham, Birmingham B15 2TH, UK.*
[d] *School of Computer Science, University of Birmingham, Edgbaston, Birmingham B15 2TT, UK.*

Materials and Methods

All chemicals and solvents used were purchased from Merck, Thermo Fisher, VWR International, Alfa Aesar or TCI. Kynurenine was purchased from Cayman chemical company. All water used was ultrapure water from a Milli-Q system. Disposable cuvettes used were made of either polystyrene (PS) or Poly(methyl methacrylate) (PMMA) and were purchased from Thermo Fisher. Greiner Bio-One black, flat bottom, 96 well microplates were used for fluorescent microplate experiments. TLC was carried out on aluminium plates coated with silica gel 60 F254 (Merck) and visualised using either potassium manganate or ninhydrin. Nuclear Magnetic Resonance spectra were recorded on a Bruker AVIII400 (400 MHz) at 298 K. Data was processed using MestReNova v12 (Mestrelab). Mass spectrometry samples were analysed by either a Synapt G2-S using an atmospheric solids analysis probe or a Xevo G2XS using electrospray ionization (both from Waters Ltd), with measurements taken using time of flight analysis on both systems.

Synthesis of sensor

The overall synthetic procedure is shown in figure below. The following sections go into more detail on the synthesis and the specific reaction steps carried out.

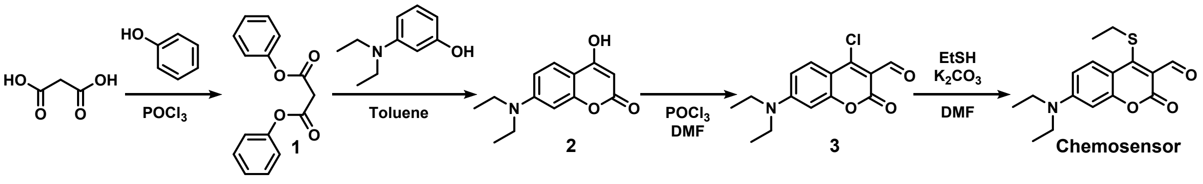

Synthesis of diphenyl malonate (1)

Following reported procedure,[1] a round bottom flask containing Malonic acid (11.0 g, 106 mmol) and Phenol (20.1 g, 213 mmol) was placed in ice bath, then $POCl_3$ (11.5 mL, 123 mmol) was added slowly. The round bottom flask was connected to a trap containing 1M NaOH aqueous solution to capture generated HCl. After stirring the mixture at 115°C for 1.5 h, HCl release ceased and two layers were formed. The upper layer was diluted with water and extracted three times with EtOAc. Combined organic phases were dried with anhydrous $MgSO_4$, filtered and evaporated to obtain **1** (21.3 g, 78.4% yield) as a pale brown oil. ^{1}H-NMR (400 MHz, $CDCl_3$) (ppm): 7.45-7.37 (m, 4H), 7.31-7.24 (m, 4H), 7.20-7.13 (m, 4H), 3.86 (s, 2H). MS (API) m/z: Calcd. for $C_{15}H_{13}O_4$ $[M + H]^+$: 257.08; Found: 257.16.

<u>^{1}H NMR</u>

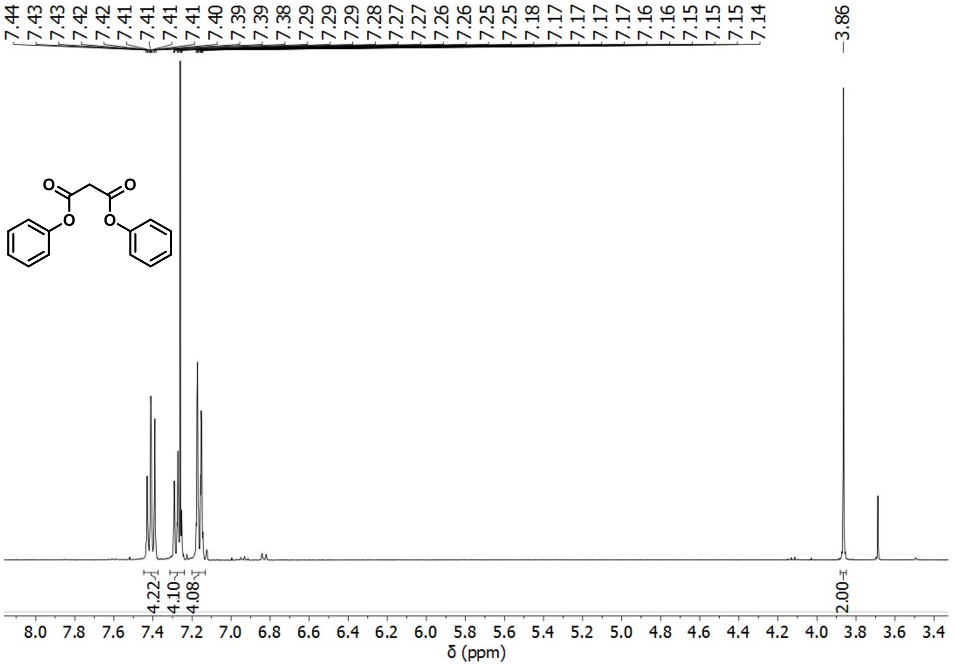

<u>MS</u>

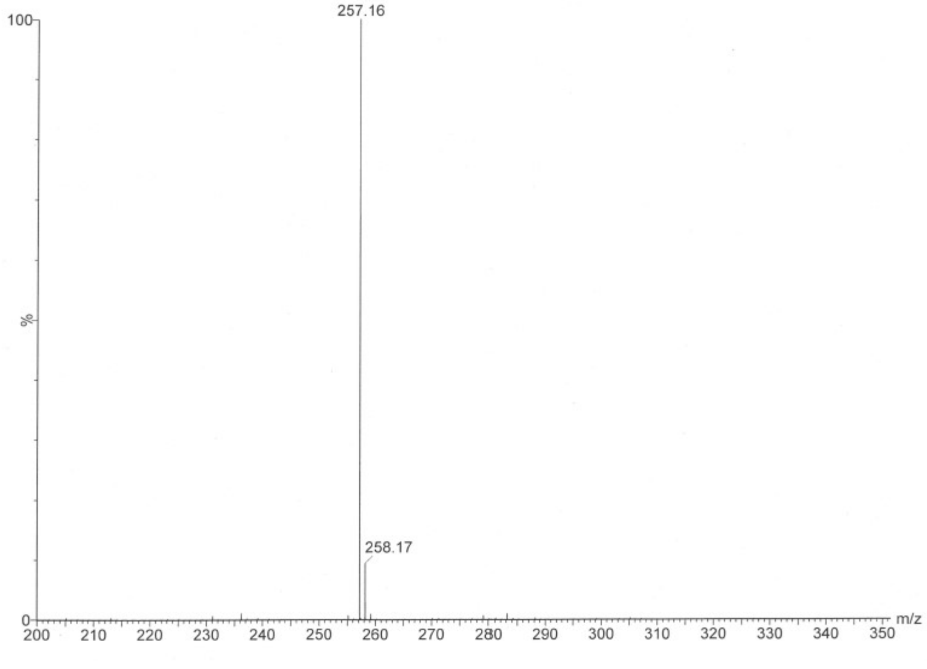

Synthesis of 4-hydroxy-7-(diethylamino)-coumarin (2)

Following reported procedure,[1] **1** (20.5 g, 80 mmol) was dissolved in toluene (80 mL), then 3-diethylaminophenol (13.2 g, 80 mmol) was added. After being refluxed for 7 h, solids in reaction mixture were filtered and washed with hexane. Once dried, coumarin **2** (6.93 g, 37.2% yield) was obtained as a light yellow solid. ^{1}H-NMR (400 MHz, DMSO-d_6) (ppm): 7.54 (d, J = 8.9 Hz, 1H), 6.65 (dd, J = 9.0, 2.5 Hz, 1H), 6.45 (d, J = 2.4 Hz, 1H), 5.25 (s, 1H), 3.40 (q, J = 7.2Hz, 4H), 1.11 (t, J = 7.0 Hz, 6H). MS (ESI) m/z: Calcd. for $C_{13}H_{16}NO_3$ $[M + H]^+$: 234.11, $C_{13}H_{15}NNaO_3$ $[M + Na]^+$: 256.09; Found: 234.1, 256.1.

<u>^{1}H NMR</u>

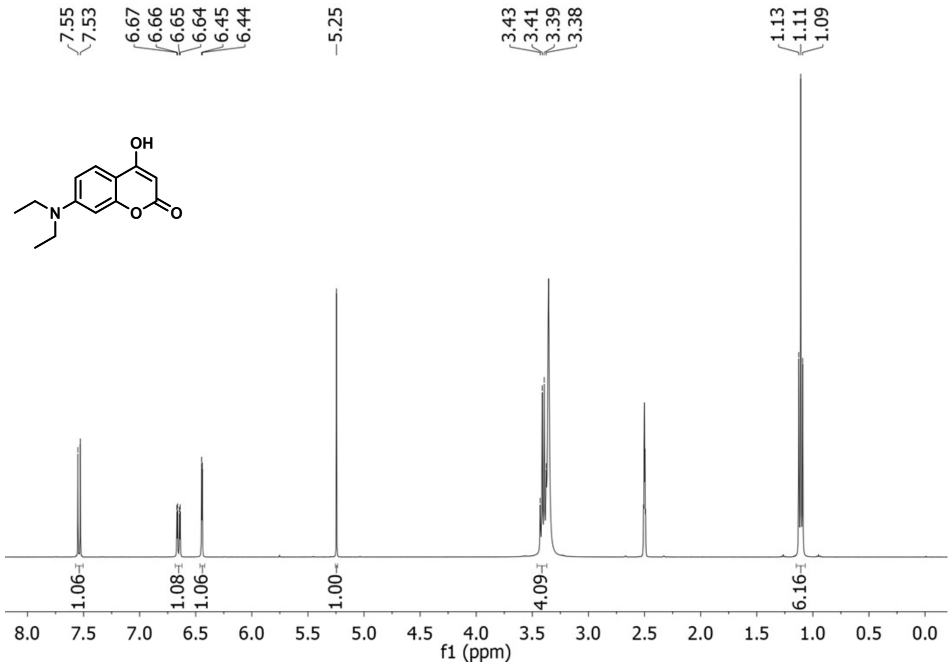

<u>MS</u>

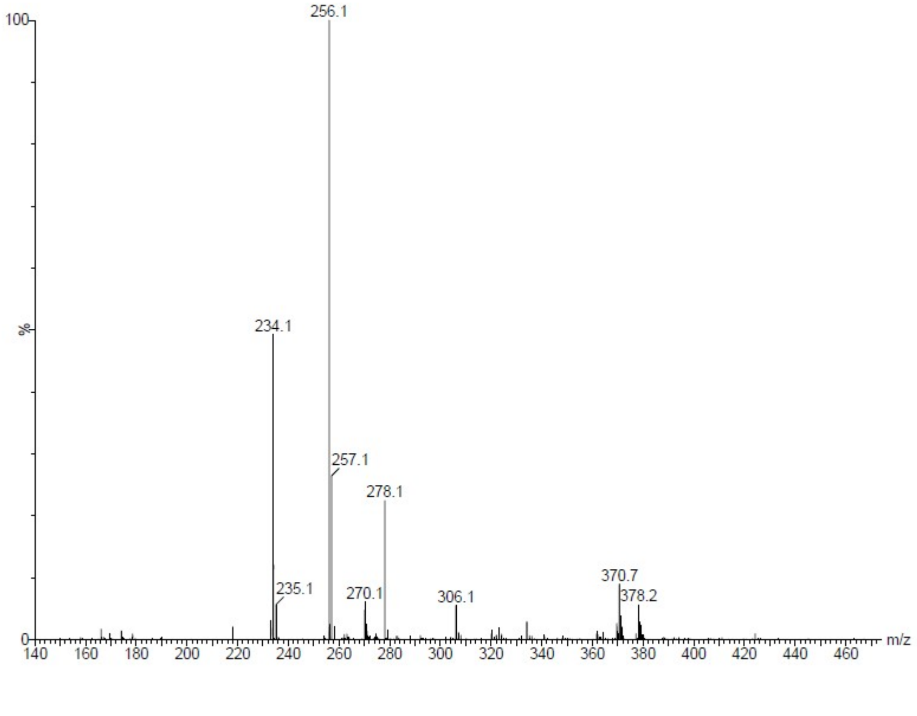

Synthesis of 3-formyl-4-chloro-7-(diethylamino)-coumarin (3)

Following reported procedure,[1] anhydrous DMF (2.8 mL) was added dropwise to $POCl_3$ (2.8 mL) in an Ar atmosphere. This solution was stirred at 50°C for 3 h, then an extra 1 mL of $POCl_3$ was added. A solution of coumarin **2** (2.33 g, 10 mmol) in DMF (13.2 mL) was added dropwise to the previous one. After stirring for 12 h at 60°C, the mixture was then poured in ice-water and its pH was set to 14 using a 5M NaOH solution. Precipitate was filtered, washed with water, dried and recrystallized with ethanol. Coumarin **3** (1.7 g, 60.9% yield) was obtained as a dark yellow solid. ^{1}H-NMR (400 MHz, $CDCl_3$) (ppm): 10.29 (s, 1H), 7.83 (d, J = 9.3 Hz, 1H), 6.69 (dd, J = 9.4, 2.5 Hz, 1H), 6.43 (d, J = 2.5 Hz, 1H), 3.48 (q, J = 7.2Hz, 4H), 1.26 (t, J = 7.2 Hz, 6H). MS (ESI) m/z: Calcd. for $C_{14}H_{15}ClNO_3$ $[M + H]^+$: 280.07, $C_{14}H_{14}ClNNaO_3$ $[M + Na]^+$: 302.06 ; Found: 280.1, 302.1.

^{1}H NMR

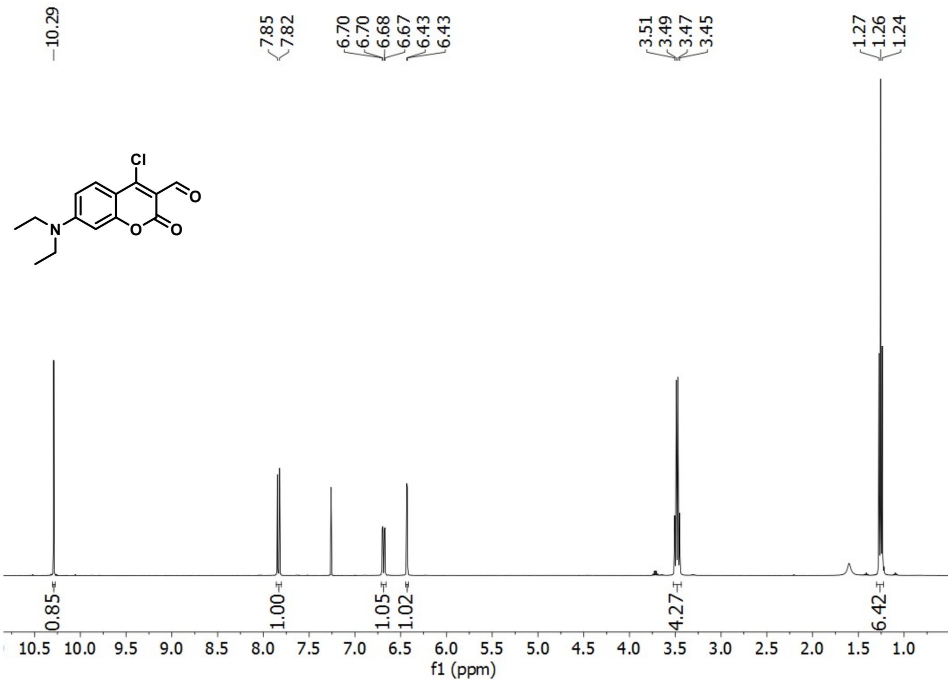

MS

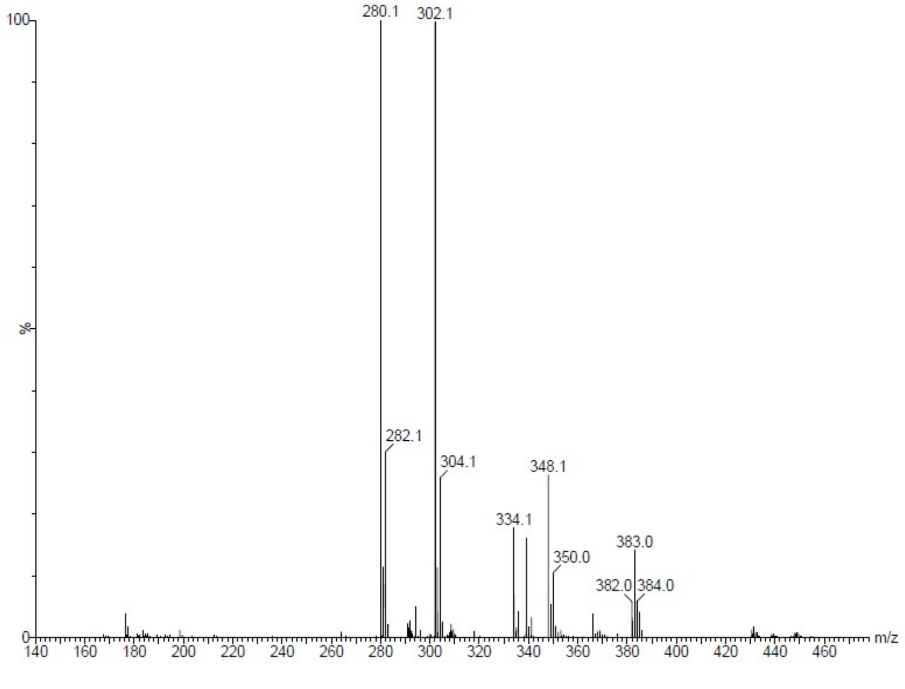

Synthesis of 3-formyl-4-(ethylthio)-7-(diethylamino)-coumarin (Chemosensor)

Following reported procedure,[2] a solution of ethanethiol (62.8 mg, 1.01 mmol) in DMF (7.0 mL) were added to a round bottom flask containing coumarin **3** (201 mg, 0.720 mmol) and K_2CO_3 (396 mg, 2.86 mmol). After stirring overnight at room temperature, reaction mixture was evaporated under reduced pressure and purified by chromatography (DCM/EtOAc 99:1 to 97:3). **Chemosensor** (75.7 mg, 34.4%) was obtained as a dark yellow powder. ^{1}H-NMR (400 MHz, $CDCl_3$) δ(ppm): 10.35 (s, 1H), 8.07 (d, J = 9.3 Hz, 1H), 6.63 (dd, J = 9.4, 2.6 Hz, 1H), 6.42 (d, J = 2.6 Hz, 1H), 3.46 (q, J = 7.1 Hz, 4H), 3.08 (q, J = 7.4 Hz, 2H), 1.28 (t, J = 7.4 Hz, 3H), 1.24 (t, J = 7.2 Hz, 6H). MS (ESI) m/z: Calcd. for $C_{16}H_{20}NO_3S$ $[M + H]^+$: 306.12; Found: 306.12.

^{1}H NMR

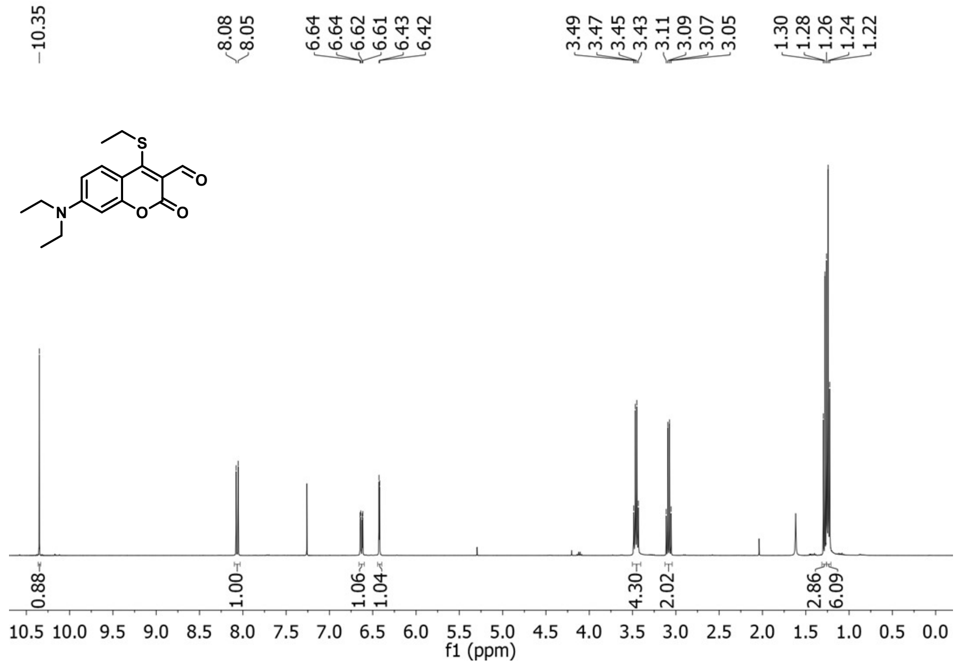

MS

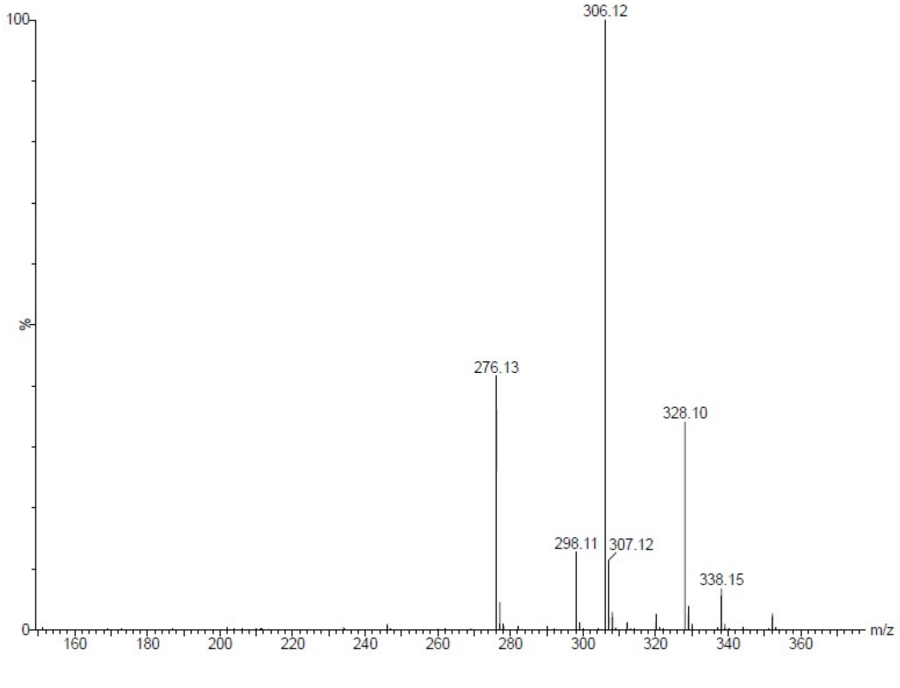

References

1 D. Song, P. Zhang, W. Guo, J. Liu, Y. Shi, Y. Huo, H. Zhang, Y.-Q. Sun and L. Wang, *J. Am. Chem. Soc.*, 2014, **136**, 574-577.

2 J. L. Klockow and T. E. Glass, *Org. Lett.*, 2013, **15**, 235-237.
